# The stock-flow model of spatial data infrastructure development refined by fuzzy logic

**DOI:** 10.1186/s40064-016-1922-1

**Published:** 2016-03-03

**Authors:** Ehsan Abdolmajidi, Lars Harrie, Ali Mansourian

**Affiliations:** Department of Physical Geography and Ecosystem Science, Lund University, Lund, Sweden

**Keywords:** Spatial data infrastructure, System dynamics technique, Fuzzy logic, Inference method, Defuzzification

## Abstract

The system dynamics technique has been demonstrated to be a proper method by which to model and simulate the development of spatial data infrastructures (SDI). An SDI is a collaborative effort to manage and share spatial data at different political and administrative levels. It is comprised of various dynamically interacting quantitative and qualitative (linguistic) variables. To incorporate linguistic variables and their joint effects in an SDI-development model more effectively, we suggest employing fuzzy logic. Not all fuzzy models are able to model the dynamic behavior of SDIs properly. Therefore, this paper aims to investigate different fuzzy models and their suitability for modeling SDIs. To that end, two inference and two defuzzification methods were used for the fuzzification of the joint effect of two variables in an existing SDI model. The results show that the Average–Average inference and Center of Area defuzzification can better model the dynamics of SDI development.

## Background

Spatial data infrastructure (SDI) is essential for successful collaborative spatial data management. SDI has been evolving and as the SDI concept matures, its complex dynamic nature is increasingly realized (Chan and Williamson 1999; Erik de Man 2006; Hendriks et al. 2012). Grus et al. (2010) addressed SDI complexity and dynamics from a complex adaptive system (CAS) point of view. Other efforts have also been made to model SDIs from different perspectives using different tools. However, most efforts to date have been limited to conceptually explaining the complexity and dynamics of SDIs (Chan et al. 2001; Erik de Man 2006; Grus et al. 2006, 2010), and fewer efforts have been made to actually model the SDI’s complexities. In fact, the better the SDI complexities are modelled, the more reliable plans can be made to develop it.

The current paper is built on a recent simulation model of an SDI development proposed by Mansourian and Abdolmajidi (2011). The authors used the system dynamics technique to model the development of an SDI by considering its dynamic and complex nature. The simulation capability of the stock-flow model of an SDI (hereafter SMSDI) enabled them to test various investment scenarios in different aspects of SDI to find the optimum policy that could lead to the further development of an SDI in their case-study area.

As with many other models, the results of the SMSDI model depend on how the factors and variables are measured and embedded in the model. This may become an issue when it involves the approximation of dynamic factors that are linguistic and vague in nature. Taking into account the characteristic of such variables and the way they are approximated or interpreted could be the main concern for improving a simulation model (Mutingi and Mbohwa 2012; Sabounchi et al. 2011; Kunsch and Springael 2008). A common way is to employ fuzzy logic (Kunsch and Springael 2008; Liu et al. 2011; Sabounchi et al. 2011). Thus, the vagueness and uncertainty of linguistic variables as another complexity in SDI development can be modeled by integrating fuzzy logic into SMSID. However, the main issue in integrating fuzzy logic for modeling multiple linguistic variables within a system dynamics simulation model is to find a proper fuzzy model. Because each fuzzification and defuzzification has its own strengths and weaknesses, there is no unique method to use. The main criterion by which to choose a model is that the fuzzy model should be able to correctly reflect the dynamic behavior of the system and the variables.

This paper aims to explore two commonly used methods of defuzzification along with two alternative inference methods. The Largest of Maximum (LOM) and Center of Area (COA) defuzzification methods were chosen. The LOM method is computationally simple and easily implemented, although it may not be the most accurate method. The COA method is more computationally complex and can more accurately reflect the continuity of fuzzy output. The Min–Max and Average–Average methods were also chosen as the inference alternatives. The Min–Max method is a popular method due to its simplicity. The Average–Average method was selected because it smooths the outputs in a dynamic environment (Sabounchi et al. 2011). Having smooth output indicates that the function has continuous derivatives.

There are several variables that can be linguistically represented in an SDI model. In this study, we selected two linguistic factors, *technological level* and *culture of data sharing* (hereafter called *level of technology* and *level of culture*), along with their joint effects on the variable *desire to participate* in SMSDI to be modeled using fuzzy logic. Expressing these variables using linguistic terms such as high, medium or low is more tangible for decision-makers. The *level of culture* and the *level of technology* also have a significant effect on organizations’ *desire to participate*. The *desire to participate* represents the inclination of organizations to participate in SDI development as a result of those influencing factors. Eventually, the behavior of the model is further studied in terms of finding and removing the counterintuitive behaviors of *desire to participate* using alternative inference and defuzzification methods.

This paper is organized in six sections, as follows. “Literature review” section, the literature review, includes a brief review of the concept of SDI, the system dynamics technique and its integration with fuzzy logic, and eventually the SMSDI and fuzzy logic as the state of the art. In “Problem statement” section, the problem is reviewed, and the necessity of this study is explained. “Fuzzy SMSDI” section, then describes the entire processes of the design and implementation of fuzzifying the SMSDI. The results and evaluation test are presented in “Results and evaluation” section. Finally, the findings of this study are summarized in “Conclusions” section, which presents the conclusion and recommendations for possible future work.

## Literature review

### SDI

The growing need to organize data across different disciplines and organizations and the need to create multi-participant, decision-supported environments has resulted in the concept of SDI. SDI refers to the infrastructure needed to facilitate the efficient and effective management, access, sharing and use of spatial data among a network of data producers/users (Hendriks et al. 2012; Hjelmager et al. 2008; Vandenbroucke et al. 2009).

SDI is dynamic and hierarchical in nature and consists of various interacting components (Rajabifard et al. 2002). In addition, a variety of institutional, technological, economic and political factors affect the development of SDIs (Crompvoets et al. 2004; Groot and McLaughlin 2000), and these factors have feedback and timely interactions. In brief, an SDI is a complex adaptive system (Grus et al. 2010) that requires a long-term process of implementation. To cope with the complexity of an SDI environment, researchers and practitioners have developed a variety of models to gain better insight into the nature and behavior of SDIs. Each model concentrates on a specific aspect of SDIs. For example, Rajabifard et al. (2002) developed a general SDI model including the core components and their mutual relationships. These authors also developed a model of the SDI hierarchy, which is comprised of inter-connected SDIs at organizational, local, state, national, regional (multi-national) and global levels. Vandenbroucke et al. (2009) then suggested a framework from a network perspective to characterize an SDI. The network model identifies the main players in a spatial data community and explains the data flow in the network of stakeholders. These models are fundamental in the way that they clarify the basic concept and nature of SDIs; based on these models, the coordinating agencies can develop their SDIs, and researchers can construct further practical and functional models.

A variety of techniques and frameworks have also been developed for the assessment of SDIs (Fernández et al. 2005; Georgiadou et al. 2006; Crompvoets et al. 2008; Grus et al. 2011). Grus et al. (2011) proposed a multi-view SDI assessment framework that provides a step-wise guideline for evaluating whether SDI implementation realizes its goals. The indicators, however, are not fixed and should be chosen from a list of possible indicators, according to some criteria.

The Commission on Spatial Data Standards of the International Cartographic Association (ICA) has defined a set of formal conceptual models for the technical characteristics of SDIs based on the ISO Reference Model for Open Distributed Processing (RM-ODP) standard. None of the above-mentioned modeling efforts addresses the direct modeling and simulation of the development process of an SDI over time. To fill this gap, Mansourian and Abdolmajidi (2011) developed a simulation model using the system dynamics technique. The model was later customized to adopt the Tanzanian SDI structure (Mansourian et al. 2015). The model developed by Mansourian and Abdolmajidi (2011) is described briefly later.

### The system dynamics technique

Sterman (2000) highlights a need for tools and processes that can help model complex systems, understand their complexity and design better operating policies. System dynamics is a technique for modeling and managing feedback systems that are complex, dynamic and nonlinear in time. This method has been used to model numerous complex systems, such as urban, industrial and ecological systems (Dudley and Soderquist 1999; Forrester 1961, 1969). The method can enhance the understanding of a complex system using simulation models, which reduce the development cost and increase the reliability of system development. A system dynamics model allows the modeler to reuse the previously built components in new system and aids in implementing the required changes (Sotaquirá and Zabala 2004).

The stock-flow model is a tool of the system dynamics technique that allows the modeler to combine qualitative and quantitative variables and calculate their feedback (Forrester 1958, 1961, 1968, 1969; Sterman 2000). It enables the modeler to simulate the system under different circumstances. In such a model, the elements of a system can be primarily modeled as stocks, flows and auxiliary variables:

- Stocks indicate the states of a system, even if the system has come to a halt. These variables are regulated over time by in- and/or out-flow variables.
- Flows control the flow into and out of the stocks. The decision is made based on the desired state of affairs, the current state of affairs, and corrective action.
- Auxiliary variables and constants are other elements that calculate or provide required information in the system.

In a stock-flow model, each variable should be modeled so that it can be included in the simulation model. Hence, the simplest model is always used to capture the characteristics of variables perceived by experts (Mutingi and Mbohwa 2012). Fuzzy logic provides better fuzzy inputs and decision rules to better reflect human judgment in the system dynamics models. This has motivated researchers in the field of system thinking to apply the concept of fuzzy system dynamics in their simulation models.

Campuzano et al. (2010) used fuzzy logic in a customer–producer–employment model in a system dynamics contest. Liu et al. (2011) also elaborately explained the implementation of fuzzy logic for modeling the combination of two linguistic variables, “delivery timeliness” and “customer service”, in a variant of a sales and service model. Neither study explained the effect of various fuzzy models on the results of their simulations. Sabounchi et al. (2011) was one of few studies to investigate alternative defuzzification methods to explore the dynamic behavior of a model. The results showed that the counterintuitive behavior of the researchers’ fuzzified model was due to the discontinuous inference methods and inconsistent rules.

As the literature denotes, few studies have been conducted to evaluate the effect of employed fuzzy models on the dynamic behavior of simulation models. Moreover, the deployed fuzzy logic in various studies highlights its context dependency in terms of defining rules and utilizing different methods of inference and defuzzification (Kunsch and Fortemps 2004; Kunsch and Springael 2008; Liu et al. 2011; Mutingi and Mbohwa 2012). Additionally, the behavior difference in crisp and fuzzy systems may vary across contexts (Mutingi and Mbohwa 2012; Polat and Bozdag 2002). In this regard, we aim to investigate the competence of different fuzzy models in modeling the joint effect of multiple linguistic variables in the SDI-development context.

### Fuzzy logic

Zadeh (1973) explains that the conventional quantitative techniques of system analysis are incompatible for addressing humanistic systems. He notes that as the complexity of a system increases, the precision of statements about its behavior leads to exclusive characteristics, and precise quantitative analyses of the behavior of a humanistic system are then likely to be irrelevant. An alternative approach, he suggests, is based on the notion that the key elements in human thought processes are not numbers but labels of fuzzy sets. The logic that plays a basic role in fuzzy theory is that the most important facet of human thinking is the ability to summarize information into linguistic characteristics that are relevant to the task at hand.

Fuzzy sets are typically used for the qualitative evaluation or comparison of systems (Piegat 2001). For instance, a manager may perceive the technological level of his/her organization as “good”, “medium” or “bad” (three classes). The first step of fuzzy modeling, called fuzzification, is to calculate the level of belonging of the current state to each class (Labib et al. 1998), which is performed using fuzzy membership functions. Taking into account the fuzzy information gathered, a user, based on his rules, decides whether to take part in the SDI development. These rules are defined as a series of condition-action statements in a fuzzy model (Kosko 1994; Labib et al. 1998). The process of making a decision is called the inference step. The aim of this step is to find the fuzzy output. There are different mechanisms of inference, such as the Mamdani implication (Min–Max), the Larsen inference method (PROD–MAX; (Kecman 2001; Kosko 1994) and Average–Average used in the dynamic systems context (Sabounchi et al. 2011). The output from this step is also the grade of membership to the output classes.

The fuzzy output should then become a crisp discrete value upon which the final decision must be based. The mechanism used to calculate the crisp value is called defuzzification (Kosko 1994; Labib et al. 1998). Various defuzzification methods exist, e.g., Largest of Maximum (LOM) model, the Center of Area (COA) model and the Fuzzy Additive Model (FAM), each with its own advantages and disadvantages (Kecman 2001).

### SMSDI

**Fig. 1:**
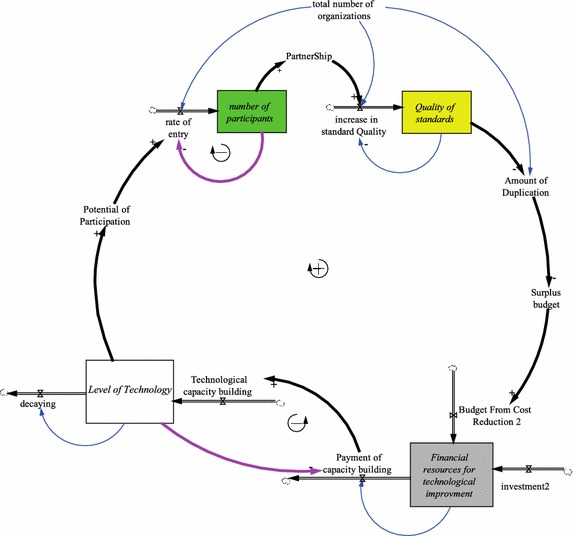
Technological growth engine (adapted from Mansourian and Abdolmajidi 2011)

**Fig. 2:**
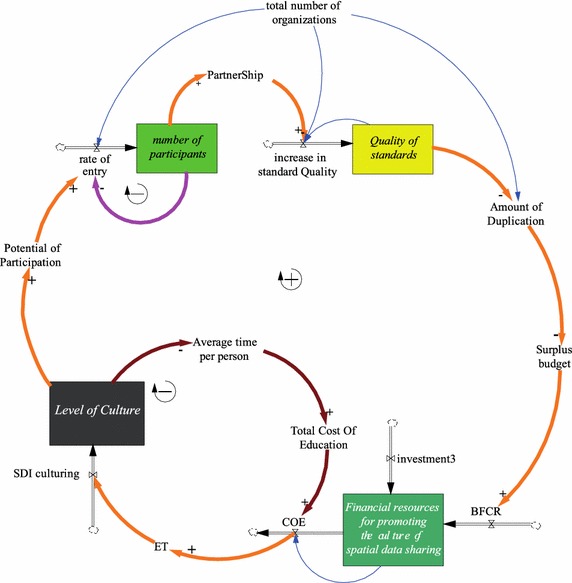
Cultural growth engine (adapted from Mansourian and Abdolmajidi 2011)

This study is based upon a model suggested by Mansourian and Abdolmajidi (2011) to simulate the development of a national SDI using the system dynamics technique in their case study area. The model embodies the major quantitative and qualitative (linguistic) factors and some of their possible relationships that affect the development of the SDI in the study area. The model includes four growth engines for *data* (production), *standard*(ization), *level of technology*, and *level of culture*. The growth engines are positive loops in a system that can continue to grow with small initial forces (Figs. 1, 2).

It is important to identify SDI growth engines and to emphasize them in SDI strategic planning because these engines help develop an SDI with a minimum amount of effort and investment. A growth-engine mechanism would exist if the benefits of the standardized up-to-date data shared in the spatial data community were returned as a financial investment in improving SDI influencing factors such as the *level of culture*, *level of technology*, human resources and standard quality. This would then consequently increase the standardized up-to-date data being shared and thus increase the benefits. In fact, the concept of SDI is the appropriate management of this procedure through the design of proper policies to maximize the standardized up-to-date shared data.

In this paper, two variables relevant to the culture and technology elements are fuzzified and studied. The growth mechanisms of these two elements are presented in Figs. 1 and 2.

Figure 1 shows the detailed structure of the technological growth engine. Mansourian and Abdolmajidi (2011) built this growth engine for modeling the improvements of the technological level of organizations and its effects on facilitating the participation of organizations in developing an SDI. Their underlying hypothesis is as follows: A superior *quality of standard*s facilitates data integration from various resources and hence has a positive effect on the sharing of spatial data. Increasing the level of data sharing reduces duplicated efforts in the production and collection of spatial data and thus reduces the associated expenses. Some part of the surplus budget from cost reduction can be used to improve the level of technology (*financial resources for technological improvement* in Fig. 1). A higher technological level increases the potential of participation in SDI development; therefore, the *rate of entry* to the SDI will grow. Finally, *increasing the number of participants* for the development of an SDI has the effect of promoting the quality of standards (Mansourian and Abdolmajidi, 2011).

The cultural growth engine (Fig. 2) works on improving the cultural aspect of SDIs. The cultural growth engine is designed to explore the effect of the *level of culture* on the development of SDIs in a spatial society. In this study, the *level of culture* represents the awareness level of the role players in SDI development about the benefits of (1) sharing data and (2) developing SDI in facilitating the management and sharing of such data. The growth engine implies that the aforementioned surplus budget can also be partially spent on the *level of culture*. By promoting a culture of data sharing and SDI awareness, increased support and incentive for participation in the development of an SDI will be achieved. A greater level of participation further reduces duplicated efforts in spatial data production (Mansourian and Abdolmajidi 2011).

As Figs. 1 and 2 show, both cultural and technological factors affect the potential of participation, which then influences the *rate of entrance* of organizations to SDI development. The *potential of participation* variable calculates the joint suitability of all influencing factors and provides the percentage of overall suitability of the system for participation. In other words, the *potential of participation* is the readiness of organizations for joining SDI development. For example, if the *potential of participation* is 10 %, then 10 % of organizations are ready to join. Hence, as the potential for participation increases, so does the rate of entrance.

## Problem statement

The factors that motivate organizations/institutes to participate in SDI development are primarily expressed by linguistic terms such as high, medium or low. For instance, an organization would be highly motivated if its level of technology were high enough and it had a high level of culture for sharing data. To better model such linguistic factors and their joint effect on the *desire to participate* in the SMSDI, we aim to use fuzzy logic in this study. The fuzzy representation of linguistic variables can better capture the characteristics of the variables and imitate the process of human decision making based on the joint effect of such variables.

Fuzzy logic is employed in different system dynamics models. Different fuzzy models, as a result of the combination of the methods used for the inference and defuzzification, can be used to fuzzify a system dynamic model. However, the fuzzified systems modeled by different fuzzy models may show counterintuitive behaviors. Sabounchi et al. (2011) believe that inconsistency in defining rules (at the inference stage) is one of the sources of the unreasonable behavior of the model. However, the rules’ inconsistency may be justifiable based on the unavoidable reality of the context, e.g., SDI. In fact, we argue that this is the nature of system when it addresses human reasoning, so we may need to find a proper fuzzy model that can better handle the seemingly inconsistent rule definitions of a fuzzified system.

## Fuzzy SMSDI

**Fig. 3:**
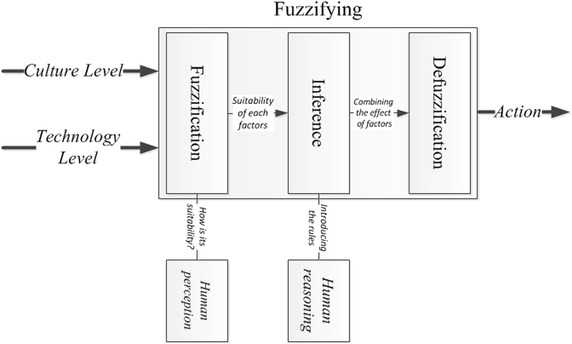
Fuzzification process in the SMSDI

The joint effect of two linguistic variables, the *level of culture* and the *level of technology*, which have a key role in attracting potential participators in developing an SDI, are modeled using fuzzy logic in SMSDI. This process is composed of the set of steps shown in Fig. 3.

The *level of culture* and *the level of technology* are two input variables in our fuzzy system. These inputs can have values of “high”, “medium” or “low” based on the interpretation of the managers of the current situation. For example, a manager looking at the current state of the culture may say that this situation is partly low or almost medium but not high. The fuzzification converts the inputs into the belonging values to each of these fuzzy sets (low, medium and high). This step is similar to the human perception of the variable status at a specific time. The output of the fuzzification is the suitability of the inputs, which is the input for the inference step.

The inference is made according to the human-defined rules resembling human reasoning for decision making. The result of the inference step is the joint effect of the factors, which is the decision being made. The decision is also a linguistic term; for instance, the “desire to participate” is expected to be “high”.

The next step is to convert this decision into action by excerpting the crisp value for the linguistic term. This step is called defuzzification.

### Design

**Fig. 4:**
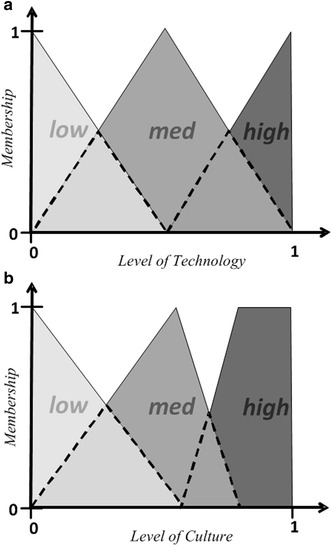
The fuzzy membership functions for **a** the level of technology of organizations: low = MFLI-Tech, med = MFMI-Tech and high = MFHI-Tech in Fig. 5, **b** the cultural level of data sharing: low = MFLI, med = MFMI and high = MFHI in Fig. 5

In the fuzzification step, we used triangular functions (Figs. 4a, b) to calculate the belonging value of the input variables to each fuzzy set during simulation. Figure 4a shows a type of linear relationship for the level of technology with its status. Because SDI development is considered a diffusion of innovation (Rajabifard and Williamson 2001; Masser 2005), awareness plays a significant role in its acceptance. It has been discussed that the awareness through time follows an S-shaped logistic curve (Kripalani et al. 2006). Hence, because the variable *level of culture* also represents the awareness of the benefits of SDI and data sharing, its improvement follows the same pattern. In other words, the *level of culture* tends to grow very slowly in the beginning of education, from a low to a medium level, with an increased growth rate. Then, it grows quickly from a medium to a high level with a decreasing growth rate. After a specific level, the influence of culture reaches the highest level. This trend is approximated by simple triangular membership functions in Fig. 4b.

The suitability of each factor is then fed to the next step, where the human decision making is resembled by the fuzzy rules and the inference method. The number of rules is determined by *C*^*L*^, where *L* indicates the number of input linguistic variables, and *C* indicates different linguistic characteristics.

Considering two input variables (*level of technology* and *level of culture*), each with three linguistic characteristics (low, medium and high), nine rules were introduced to the model (Table 1).

**Table 1:** 9 rules for the inference step

Rules	Level of technology	Level of culture	Desire to participate
R1	High	High	High
R2	Medium	High	High
R3	Low	High	Medium
R4	High	Medium	High
R5	Medium	Medium	Medium
R6	Low	Medium	Medium
R7	High	Low	Medium
R8	Medium	Low	Low
R9	Low	Low	Low

*L* low, *M* medium, *H* high membership functions

To define these rules, expert opinions were used. Experts believe, in SDI development, that culture has more effect on attracting participants than technology. This belief is reflected in rules R6 and R8. In rule 6, the level of culture is medium, and the level of technology is low. SDI experts then expect the level of *desire to participate* to be medium, whereas in the reverse situation, as in R8, the *desire to participate* is considered low. In both situations, culture has more influence and skews the output toward itself.

After the rules were defined, their values were calculated using the inference methods. Here, we employed Max–Min and Average–Average as the inference alternatives. This step can be considered human reasoning. The results of the inference step were fuzzy outputs. To be more precise, they were the values belonging to the linguistic characteristics (high, medium, and low level of *desire to participate*) of the domain *desire to participate*.

At the last stage, fuzzy output was converted to a crisp value that determined the state of action that should take place; i.e., the *desire to participate* determined the percentage of participation. To extract the crisp value, the Largest of Maximum (LOM) and Center of Area (COA) defuzzification models were used.

### Implementation

**Fig. 5:**
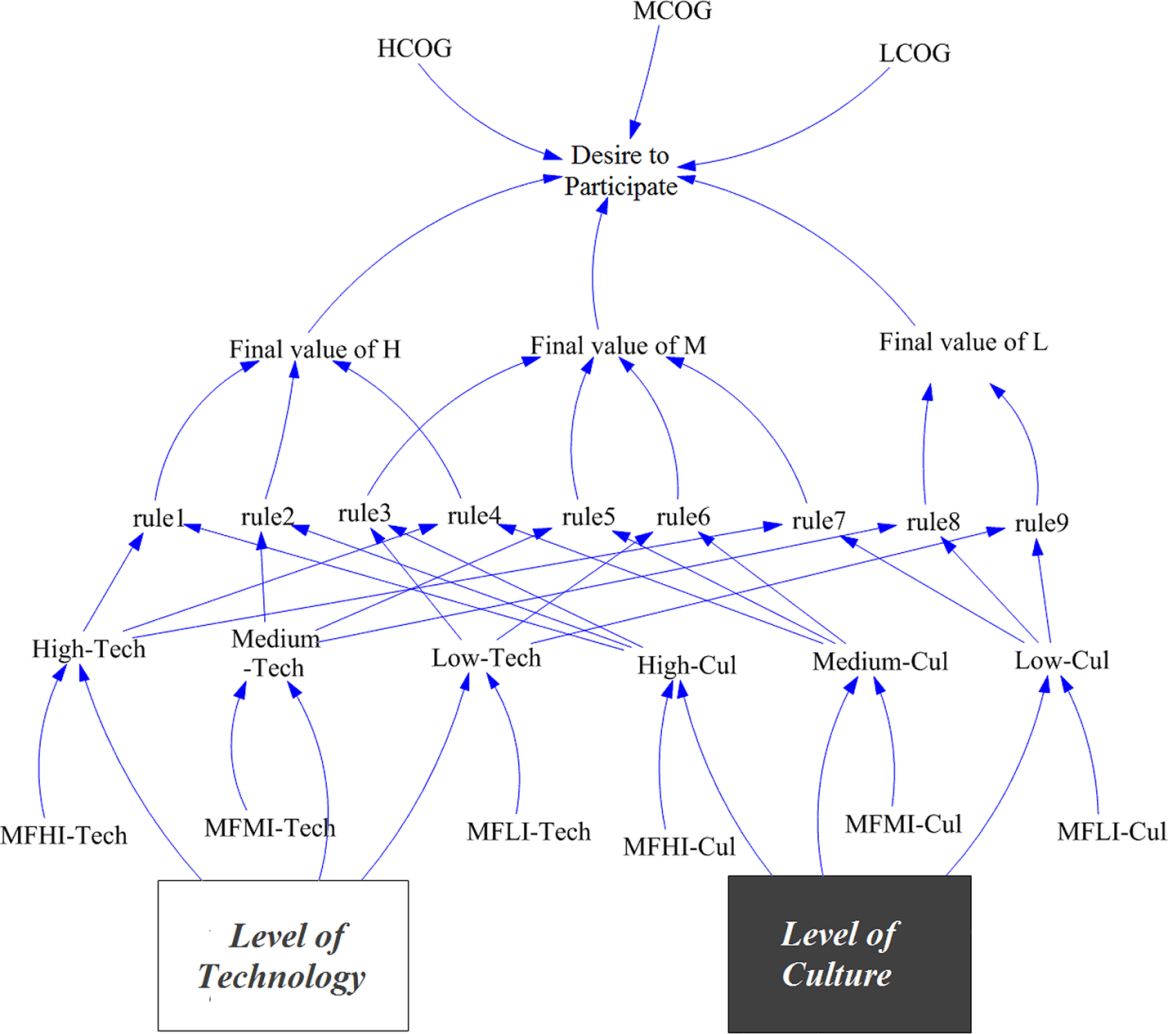
The fuzzy structure for modeling two linguistic variables: 1) *level of culture* and 2) *level of technology* in SMSDI. *MFHLI*, *MFMI* and *MFHI*-*tech* and -*Cul* represent the fuzzy membership functions of *level of technology* and *level of culture*

The model was developed in Vensim PLE version, which is free for educational research. In the evaluation step, the first and second tests discussed were conducted in Vensim and Stella, respectively. Figure 5 shows the stock-flow structure of the entire fuzzifying process, replaced with the corresponding variables and structure in the SMSDI.

As Fig. 5 depicts, the fuzzy structure has several variables that are responsible for the aforementioned steps of fuzzifying (fuzzification of inputs, fuzzy inference, and defuzzification). The variables belonging to each of these steps are explained in the following sections.

#### Fuzzification of inputs

Three linguistic characteristics, *high*, *medium* and *low*, were considered for each input variable. In Fig. 5, *MFHLI*, *MFMI*, and *MFHI*-*Tech* are look-up tables representing the low, medium, and high membership functions of the technological level, respectively. For the level of culture, each characteristic was modeled by the *MFHLI*, *MFMI*, and *MFHI*-*Cul* lookup tables. The belonging values of the input variables to each characteristic were then calculated. Equation 1 exemplifies the calculation of the belonging value of the *level of technology* at time t_i_ to the High_Tech fuzzy set [µ_ℎ − Tech_(t_i_)] according to the MFHI_Tech fuzzy membership function.

1 $$ \upmu_{{High} - {\rm Tech}} \left( {{\text{t}}_{\text{i}} } \right) = {\text{MFHI\_Tech}}\left( {{\text{Technological level of organizations}}\left( {{\text{t}}_{\text{i}} } \right)} \right) $$

In other words, using Eq. 1, we determined how much the *level of technology* (at time t_i_) belonged to the High-Tech fuzzy set. Similar equations were also used for the other fuzzy sets. A High-Tech membership value of one indicated that organizations reached the highest level of technology required for SDI development.

#### Fuzzy inference

**Fig. 6:**
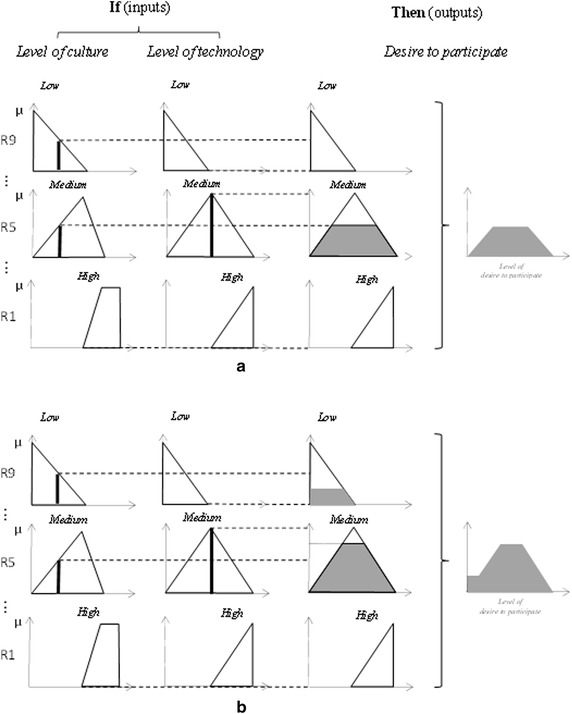
Fuzzy inference methods: **a** Max–Min and **b** Ave–Ave inference methods. A snapshot of fuzzy inference at time t = 0.625 year (*black solid line*)

A fuzzy inference resembles the human reasoning over the variables (here, *level of culture* and *level of technology*) for decision making. It consists of several steps for mapping fuzzified input(s) to fuzzy output. The first step was to define the inference rules. The defined rules in Table 1 were explained in the form of “IF… THEN…” statements, constituting two inputs and one output. An example rule (R7) is “*IF level of culture is low AND level of technology is high, THEN desire to participate is medium*”. It was observed that the relationship between two inputs was a logical “AND”. This relationship could be calculated by any T-norm operator, such as the MIN or average operators (Kecman 2001). In this research, we first used the min operator and then substituted it with the average operator. For the min operator, the minimum belonging values of inputs was extracted for rules R1 to R9 (Fig. 6a). The average operator, however, calculated the mean value of the inputs (Fig. 6b). These values represented the belonging value of the output membership functions defined by those rules (Fig. 6).

For instance, in Fig. 6, the input and the output values of rule R9 at time t (=0.625 year) are shown for both the Max–Min (Fig. 6a) and Ave–Ave (Fig. 6b) inference methods. The belonging values of “low” *level of culture* and “low” *level of technology* are 0.37 and 0.00, respectively, at the given time for both inference methods; however, the output belonging value varies for these two methods. The Max–Min output value of a “low” *desire to participate* is 0.00, and the Ave–Ave output value is 0.185. These calculations take place in R1 to R9 variables in Fig. 5.

The last step in the fuzzy inference is to evaluate all rules together using a union operator. In this study, we used two alternative operators: max and average. These operators calculate the final belonging value for each domain (output) fuzzy set (low, medium and high level of *desire to participate*). The max operator chooses the maximum value of the rules’ outputs, whereas the average operator calculates their mean value. These values are computed in three variables, i.e., “final value of H”, “final value of M”, and “final value of L” (see Fig. 5).

The Max–Min inference, known as Mamadani’s method (Mamdani 1977), is the most common inference method, and Average–Average is recommended for use in dynamic models to smoothen the inference outcomes.

### Defuzzification

In the defuzzification step, the final crisp value should be calculated so that the final action can take place. There are various defuzzification methods; each has its own strengths and weaknesses. In dynamic modeling, a modeler is primarily concerned with the dynamic behavior of the model and the constituting variables. Therefore, a defuzzification method is suitable to retain this property. With this in mind, we chose to employ two defuzzification methods, Largest of Maximum (LOM) and Center of Area (COA). The LOM method is easy to implement, whereas the COA has smoother output.

**Fig. 7:**
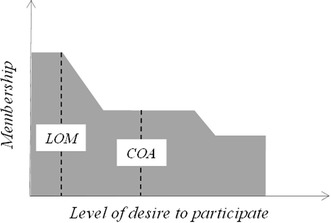
LOM and COA defuzzification

The defuzzification process takes place in the variable *desire to participate* (Fig. 5) using the inference outcomes (grey area in Fig. 7) as inputs. The lookup tables LCOG, MCOG, and HCOG (see Fig. 5) respectively represent the membership functions of domain characteristics *Low*, *Medium*, and *High*.

The LOM method returns the largest value that can produce the maximum belonging value to the relevant membership function of the domain (Fig. 7). The COA method is the weighted mean (Eq. 2) of the grey area in Fig. 7.

2 $$Z_{0} = \frac{{\int {\mu_{x} xdx} }}{{\int {\mu_{x} dx} }}$$

where *μ*_*x*_ is the membership value for each characteristic, and x is the domain value in the membership function for that membership value. We approximated Eq. 2 with Eq. 3, where x is the minimum value producing *μ*_*x*_ in that member function.

3 $$Z_{0} = \frac{{\sum {\mu_{x} x} }}{{\sum {\mu_{x} } }}$$

## Results and evaluation

**Fig. 8:**
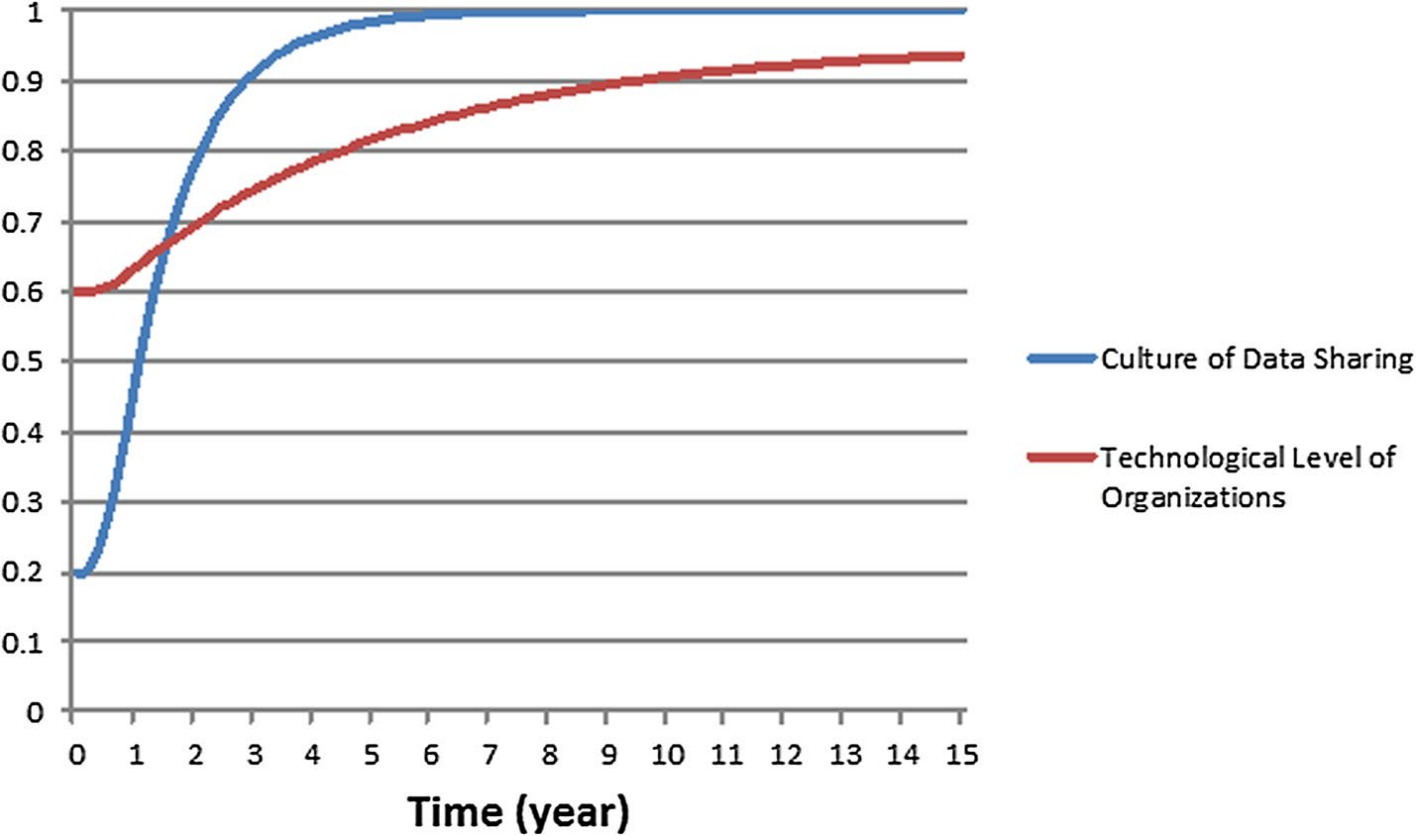
Improvement in the level of culture and level of technology during the years SDI was developed

To evaluate the fuzzy models, two tests were considered: behavior reproduction and multivariate sensitivity analysis. These tests are the most popular tests in system dynamics simulation models because the simulation models are expected to behave reasonably according to the inputs behavior. Behavior reproduction is selected to assess the ability of the model in imitating the dynamic behavior of the real system according to the behavior of the inputs. Figure 8 shows the behavior of the two inputs of the fuzzy models: *level of culture* and *level of technology*. According to Fig. 8, it is expected that the *desire to participate* grows as the *level of culture* and *technology* improves over the years. If there is any counterintuitive behavior, the possible reasons and solutions should be investigated and discussed.

The multivariate sensitivity analysis then investigates the sensitivity of the *desire to participate* variable to various combinations of inputs values (the level of culture and level of technology). This test can confirm that the model is consistent for any possible value.

### Behavior reproduction test

**Fig. 9:**
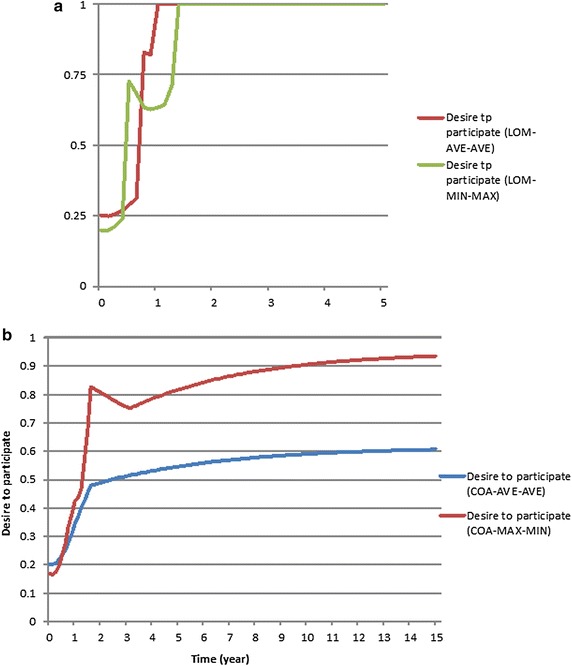
Behavior of the variable *desire to participate*; **a** the LOM defuzzification coupled once with Max–Min and once with Average–Average inference methods and **b** the COA defuzzification coupled once with Max–Min and once with the Average–Average inference method

Figures 9a, b demonstrate the effect of four fuzzy models on the behavior of the variable *desire to participate*. At time 0.375 in LOM-MAX–MIN (Fig. 9a), a discontinuity can be observed; the variable’s value suddenly increases from 0.237 to 0.728. Then, the variable’s value starts to decrease, though it is expected to increase as time passes.

As Fig. 9a shows, these two fuzzy models cannot reflect the expected consistently growing behavior for the desire to participate. The first reason for the counterintuitive behavior is primarily due to the defuzzification method used here. When the *desire to participate* transitions from low to medium, there is a sudden increase in the final crisp value as the LOM method returns the largest value in the medium level. This explains the discontinuity in the behavior of LOM-MAX–MIN and LOM-AVE–AVE. Additionally, when the value belonging to the medium level of the *desire to participate* increases because of the improvements in the inputs, the largest of the medium level declines. This also explains the decrease in the *desire to participate* for the LOM-MAX–MIN and LOM-AVE–AVE models in Fig. 9a.

The second reason is due to the definition of the rules. Rules 6 and 8 (see Table 1) have the same antecedents with varying consequences. Rule 6 has a low technological level and a medium level of culture, resulting in a medium *desire to participate*. Rule 8, with a medium level of technology and a low cultural level, results in a low level of desire to participate. Experts have defined these rules in this way because they believe that culture has a higher influence than technology on attracting potential role players to participate in SDI development. Hence, the higher cultural level leads to a higher desire for participation. It is the nature of human reasoning to deem some factors more influential than others, leading to common inconsistency in defining rules. Some researchers have suggested correcting this rule inconsistency to obtain a reasonable dynamic behavior (Sabounchi et al. 2011). We, however, argue that there may be a proper fuzzy model that can handle rule inconsistency, which is a part of human nature and context dependent. Therefore, we can conclude that the LOM-MAX–MIN and LOM-AVE–AVE fuzzy models (Fig. 9a) cannot handle the rule consistency in SMSDI in the current setting.

Figure 9b shows the behavior of the variable *desire to participate* for the other two fuzzy models. The COA-MAX–MIN fuzzy model in Fig. 9b also exhibits counterintuitive behavior from time t_1_ = 1.625 to t_2_ = 3.750. The investigation shows that from t_1_ = 1.625, the *desire to participate* is actively influenced by the variable *final value of H*, which also shows behavior similar to the *desire to participate*. This finding shows that the Max–Min inference method cannot smoothly combine rules R1, R2, and R4. However, the COA-AVE–AVE fuzzy model shows a reasonable behavior for the *desire to participate* (Fig. 9b). This finding shows the influence of a smoother inference method, such as Average–Average, on the dynamic behavior of the fuzzy model.

### Sensitivity analysis

To assure that the behavior of the fuzzy models was consistent in any combination of fuzzy input values, the second test, the multivariate sensitivity analysis, was individually applied to the fuzzy structure for the COA-Average–Average model because it was the only fuzzy model that passed the behavior-reproduction test.

**Fig. 10:**
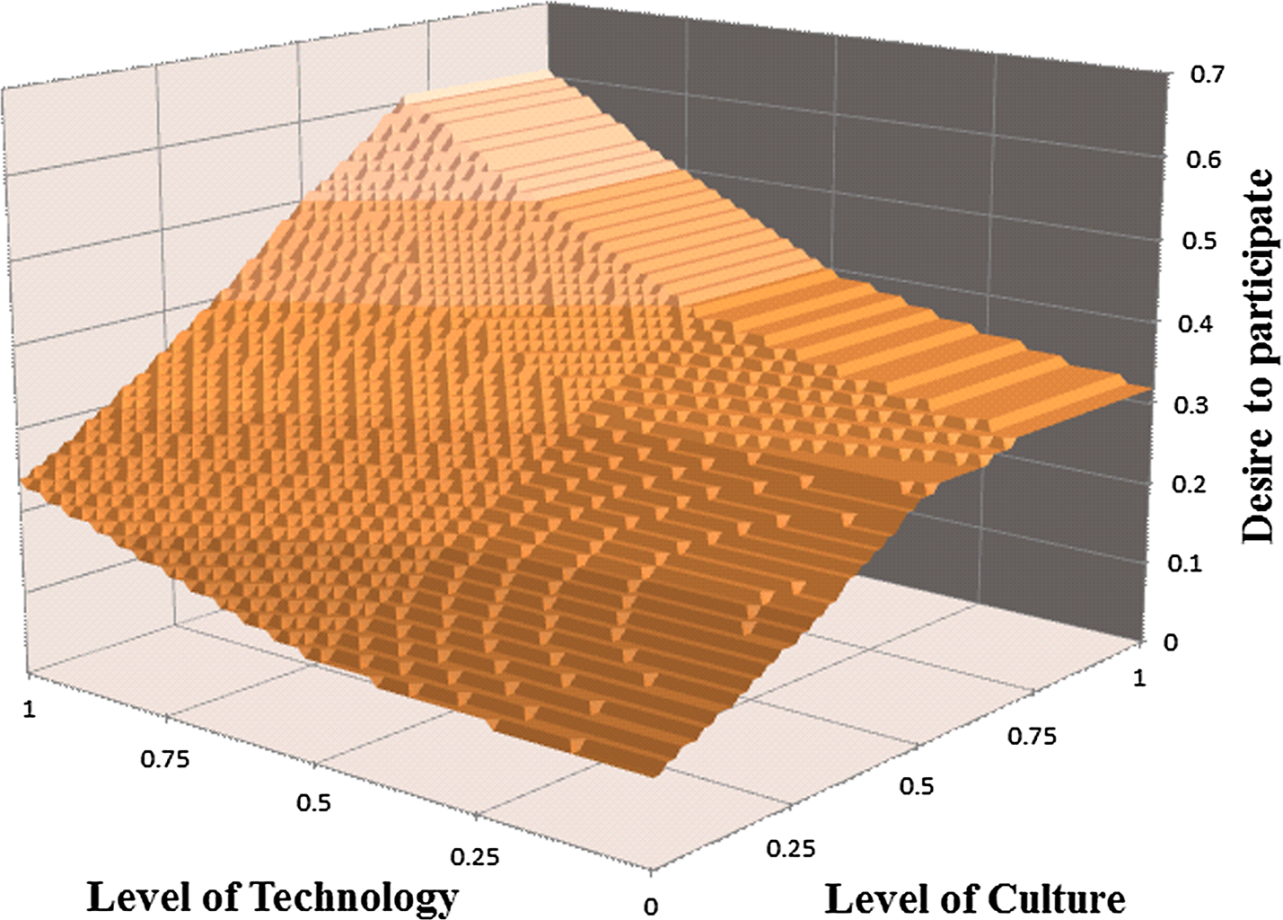
Multivariate sensitivity analysis of fuzzy model COA-Average–Average

Figure 10 shows the result of the multivariate sensitivity analysis for the output variable *desire to participate* using the COA-AVE–AVE fuzzy model. The behavior of the *desire to participate* is plotted against values of input variables, *level of culture* and *level of technology*, ranging from 0 to 1. As expected, the *desire to participate* at point (level of culture, level of technology) = (1, 1) has the maximum value. That is, when organizations have the highest *level of technology* and *culture*, it is expected that their *desire to participate* will be at the highest level. As the situation changes simultaneously on both axes of *level of culture* and *level of technology* toward zero, the desire should also decrease significantly to the minimum value. As Fig. 10 shows, the behavior of the output, from the best situation to the worst situation, decreases in the COA-Average–Average model.

Additionally, when the *level of technology* is at the minimum level, moving down the *level of culture* axis shows that the *desire to participate* starts to increase from the minimum and keeps growing until the *level of culture* has its highest effect on the level of *desire to participate*. The *desire to participate* does not achieve the maximum value because of the lack of technological capacity. The technological limits of organizations appear to be the reason for unwillingness to participate in SDI development.

Moving down the *level of technology* axis, when the *level of culture* is at the minimum level, the *desire to participate* also consistently grows. In this case, the desire does not reach to its highest level because the cultural factor is lacking. Moreover, it is worth highlighting that the highest *desire to participate* in this case is less than that in the case where the technological capacity is neglected. Therefore, the model can properly reflect the importance of the culture in provoking the *desire to participate*, which we tried to take into account in defining the rules according to the experts. Thereafter, the results show the consistency in the behavior trend. This consistency comes with a price of counterintuitive behavior in extreme situations. The value of *desire to participate* when the values of the pair (*level of culture*, *level of technology*) are (0,0) and (1,1) should be the lowest (0) and highest (1), respectively. However, its values are, respectively, 0.1 and 0.61, due to the averaging nature of COA.

## Conclusions

Fuzzy logic can be integrated with system dynamics models to interpret the current status of various qualitative factors in a way that is more readily understood and applied for the key players in a system. With this approach in mind, we suggest redefining the modeling of the joint effect of linguistic variables on the SMSDI using fuzzy logic because the model involves several linguistic variables. However, considering unavoidable inconsistencies in rule definitions of SDI and the incompatibility of the fuzzy models with the dynamic systems, it is required that a proper fuzzy model be used that can model the dynamic behavior of SDI in SMSDI.

We investigated four fuzzy models to model the joint effect of two linguistic factors, *level of culture* and *level of technology*, in the simulation model of developing SDI (SMSDI). A different combination of two inference methods, Max–Min (Mamdani) and Average–Average, and two defuzzification methods, Largest of Maximum (LOM) and Center of Area (COA), were studied.

A new fuzzy structure was added to SMSDI to model the joint effect of two influencing factors. Afterward, different fuzzy models were applied and evaluated by the *behavior reproduction* and *sensitivity analysis* tests. The results showed that the COA defuzzification coupled with Average–Average inference could reflect better than the other three models the dynamic behavior of SDI development. However, this model behaved counterintuitively at extreme points, due to the averaging nature of the COA defuzzification. This may raise the necessity of investigating more fuzzy models in future studies.

The other models used here primarily suffer from discontinuity by considering the maximum or minimum values in inference and defuzzification steps. The other reason for their counterintuitive behavior is the inconsistency in defining the rules, which could be justifiable in the SDI development context. Nevertheless, this problem may be addressed by using more linguistic characteristics for the fuzzy inputs and output, e.g., very low, low, medium, high and very high *desire to participate* in SDI development.

Considering the successful integration of fuzzy logic and the system dynamics model, the first priority of future work is to investigate more fuzzy models and to include other linguistic factors influencing SDI development in the fuzzy representation. The fuzzified SMSDI enables modelers to practice various scenarios, such as the worst-case scenario, because they can define the pessimistic rules. Moreover, they can simply change the rules to better adjust them to their own society.

## Abbreviations

SDI: spatial data infrastructure
ICA: International Cartographic Association
SMSDI: simulation model of spatial data infrastructure development
Ave-Ave: Average–Average method
COA: Centre of Area
LOM: Largest of Maximum
